# How Do We Update Faces? Effects of Gaze Direction and Facial Expressions on Working Memory Updating

**DOI:** 10.3389/fpsyg.2012.00362

**Published:** 2012-09-27

**Authors:** Caterina Artuso, Paola Palladino, Paola Ricciardelli

**Affiliations:** ^1^Department of Psychology, University of PaviaPavia, Italy; ^2^Department of Psychology, University of Milan-BicoccaMilan, Italy

**Keywords:** biological binding, social cognition, appraisal, gaze direction, facial expression, facial dimensions, working memory

## Abstract

The aim of the study was to investigate how the biological binding between different facial dimensions, and their social and communicative relevance, may impact updating processes in working memory (WM). We focused on WM updating because it plays a key role in ongoing processing. Gaze direction and facial expression are crucial and changeable components of face processing. Direct gaze enhances the processing of approach-oriented facial emotional expressions (e.g., joy), while averted gaze enhances the processing of avoidance-oriented facial emotional expressions (e.g., fear). Thus, the way in which these two facial dimensions are combined communicates to the observer important behavioral and social information. Updating of these two facial dimensions and their bindings has not been investigated before, despite the fact that they provide a piece of social information essential for building and maintaining an internal ongoing representation of our social environment. In Experiment 1 we created a task in which the binding between gaze direction and facial expression was manipulated: high binding conditions (e.g., joy-direct gaze) were compared to low binding conditions (e.g., joy-averted gaze). Participants had to study and update continuously a number of faces, displaying different bindings between the two dimensions. In Experiment 2 we tested whether updating was affected by the social and communicative value of the facial dimension binding; to this end, we manipulated bindings between eye and hair color, two less communicative facial dimensions. Two new results emerged. First, faster response times were found in updating combinations of facial dimensions highly bound together. Second, our data showed that the ease of the ongoing updating processing varied depending on the communicative meaning of the binding that had to be updated. The results are discussed with reference to the role of WM updating in social cognition and appraisal processes.

## Introduction

Gaze direction and facial expression are crucial components of face processing, particularly because they communicate the intentions of others, and, in general, being able to read the mind from the face is important not only for social interaction but is also advantageous for navigating in a social environment. Previous studies demonstrated that the influence of gaze direction on the perception of human faces varies depending on the behavioral intention associated with the facial emotion that is expressed (e.g., Adams and Kleck, 2003; Conty et al., 2012). Direct gaze enhances the perception of approach-oriented facial emotional expressions, such as anger or joy, while averted gaze enhances the perception of avoidance-oriented facial emotional expressions, such as fear or sadness. Indeed, facial expression and gaze direction are associated to signal these basic behavioral tendencies and their processing appears to be combined.

Appraisal theories of emotion (e.g., N’Diyae et al., 2009; Mumenthaler and Sander, 2012) emphasize the fact that the combination of gaze direction and facial expression indicates to the perceiver the degree of self-relevance of the seen face. For instance, for survival, the self-relevance of a threat signaled by a fearful face should increase when the face is looking at something away from the observer. This is because the averted gaze signals where in the environment a threat may come from. Whereas the opposite should be true for an angry face looking straight at the observer since the direct gaze indicates impending aggression toward the observer him-herself. Therefore, the appraisal of the face also provides important social information to be processed and then to be taken into account.

Not only are facial emotional expressions and gaze direction included in the evaluation process to determine the behavioral intention of the other person and the relevance to oneself, but their bound processing appears to occur rapidly and automatically, as Milders et al. (2011) demonstrated through an attentional blink task. In fact, they found that specific emotions were detected more frequently when associated with specific gaze directions. Namely, fearful faces were detected more frequently with averted than with direct gaze, whereas angry and happy faces were detected more frequently with direct than with averted gaze. Similarly, Ricciardelli et al. (2012) found that angry faces displaying direct gaze produced no attentional blink, indicating that such a threatening combination of facial expression and gaze direction was processed fast and automatically, probably by virtue of its high social and adaptive value.

Therefore, there is evidence, from the domain research of perception, emotion and attention, of an enhanced processing of specific and highly communicative combinations of gaze direction and facial expression. Moreover, there is also some evidence of the influence of gaze and facial expression on face memory. In particular, poorer memory for angry faces that were initially presented with averted gaze as opposed to direct gaze has been reported (Nakashima et al., 2012). The appraisal of a face (i.e., deceptive faces) influences its encoding in the memory (e.g., Yamagishi et al., 2003; Bayliss and Tipper, 2006). Surprisingly however, it is unknown whether and how the highly communicative combinations of gaze direction and facial expression affect working memory (WM), a function of primary importance for ongoing processing and for dealing with either environmental stability or flexibility (e.g., Kessler and Meiran, 2008).

Working memory is a dynamic cognitive system and it is likely to work largely through the mechanism of updating which is responsible for the fast processing and appraisal of information useful to our current goals. Updating can be conceptualized in terms of the maintenance and inhibition of information, since its function is to maintain goal relevant information and to inhibit information that is irrelevant (see Morris and Jones, 1990; Palladino et al., 2001; Oberauer, 2005). Indeed, this function of updating should be especially observed and important when we are engaged in processing social salient stimuli such as faces. In fact, in order to read the social meaning of a face, we have to quickly select relevant information from it and, when needed, to update it. Particularly, this is true for the information coming both from facial expression and gaze direction which represent highly communicative and changeable aspects of faces (see Haxby et al., 2000). Thus, when living in a social world, being able to quickly detect changes in faces and to update them is undoubtedly beneficial in terms of the ability to evaluate the face and understanding its meaning. The WM updating mechanism should be certainly involved in all these social cognitive abilities but it has not been studied using face as stimulus.

Recently, basic research in WM has addressed the role of binding (or association/combination) as an important factor in explaining performance. Moreover, the ability to build-up, maintain, dismantle, and recreate a binding has been considered a primary source of individual and age-related differences (see Oberauer, 2005; Schmiedek et al., 2009). Bindings may also represent different types of relational memories: for example, between stimuli and their features (e.g., item-color) or between unrelated stimuli that have highly similar characteristics (e.g., item–item, see Piekema et al., 2010).

A recent study by Artuso and Palladino (2011) investigated how bindings may affect the updating process in WM. Specifically, they manipulated the arbitrary association between two consecutive items, i.e., letters; for example, if *BFC* is a set of items, a binding could be the combination between *B* and *FC*, or *BF* and *C*. They created a computerized task where they manipulated the strength of the binding through the perceptual similarity with which the stimuli were bound together. They found that stronger bound perceptual configurations (i.e., stimuli depicted using identical colors), needed longer latencies to be updated, compared to weaker bound configurations (i.e., stimuli depicted in different colors; for details see Artuso and Palladino, 2011, see Results *On-line: Perceptual binding*). The finding was similar to that found with stimuli characterized by phonological similarity (Guérard et al., 2009). Altogether, these results show that WM performance is affected by the strength with which stimuli are bound together by some kind of similarity (e.g., perceptual or phonological), at least for stimuli with no, or very little, biological relevance.

A relevant methodological aspect of the Artuso and Palladino’s (2011) study was that in the task they measured on-line response time (RT), an alternative way to assess WM thought to represent a more sensitive index of the process occurring during the task, thus enabling a more direct and clear-cut measurement of the updating process (e.g., Kessler and Meiran, 2008; see the Method and the Results for further details). In the study by Artuso and Palladino (2011) trials were composed of steps requiring the performance of different cognitive operations, i.e., studying, maintenance, or updating of information. Interestingly, the authors showed that on-line RT was useful to track the updating process: participants’ RTs were longer in updating steps, when compared to either maintenance or studying steps. This supported the view that updating is much more effortful, relative to simple memory maintenance of information (see Results *On-line: Updating effects*; see also Palladino and Jarrold, 2008).

It’s worth noting that all of the aforementioned studies used high bound stimuli, but with no biological and communicative value; in other words, arbitrarily created stimuli. Further, such stimuli combinations do not have a role of primary importance either in terms of ongoing behavior or in terms of appraisal processes of the stimulus. We believe that the two dimensions of gaze direction and facial expression can be reasonably considered and treated as a special kind of binding, i.e., a biological binding. This is because of their biological, social, and communicative value, and due to the fact that they are preferentially processed in combination (e.g., Adams and Kleck, 2003; Conty et al., 2012). Therefore, the updating of these bindings should be particularly meaningful in a social environment. Thus, respect to stimuli with no explicit biological value (such as letters or digits), they might have a different impact on WM updating. We believe, in fact, that faces represent one of the main stimuli whose processing undoubtedly contribute to building-up and maintaining an internal model of what has been, and is happening in our social world. This is analogous to what has been reported for the on-line maintenance and manipulation of other pieces of social information (e.g., thinking about the psychological characteristics of people, see Meyer et al., 2012). Consequently, the updating of crucial and communicative components of the face (such as gaze direction and facial expression) is necessary to handle representations of the immediate social environment.

In the present study, we tested the impact that biological bindings may have on WM updating. By biological binding we mean different combinations between facial dimensions.

Our aim was twofold. First, to investigate whether when stimuli have a biological value, the strength of their bindings was detrimental or beneficial on updating performance. In particular, in Experiment 1 we regarded as high biological bindings the combinations between approach-oriented facial expressions (i.e., joy) and direct gaze and those between avoidance-oriented facial expressions (i.e., fear) and averted gaze. The reason was because they were previously found to be perceived and processed strongly bound together and to have a role in appraisal processes (e.g., N’Diyae et al., 2009). Whereas, we regarded as low biological bindings those combinations in which approach-oriented facial expressions were combined with averted gaze, and avoidance-oriented facial expressions were combined with direct gaze. We hypothesized that, by virtue of their biological, social, and communicative value, the strong combinations of gaze direction-facial expression, i.e., high bindings, should be updated faster than the weak gaze direction-facial expression combination, i.e., low bindings. Therefore, we assumed that this biological binding might be beneficial to WM updating given its relevance and its function of maintaining an internal model of social environment (see also Meyer et al., 2012), and also because they are processed fast and automatically (Milders et al., 2011; Ricciardelli et al., 2012).

Second, we aimed to study whether (as we expected) the impact of biological bindings on WM updating is affected, thus varies, as a function of the social and communicative value of the facial dimensions considered. In fact, given that the combination of gaze direction and facial expression also conveys the relevance of the face for the observer (i.e., face appraisal, see e.g., N’Diyae et al., 2009), it would be possible that this piece of information also has to be dealt with by WM memory updating. To this end, we compared the biological binding between the two highly social/communicative dimensions of gaze direction and facial expression (see Experiment 1), with the biological binding between two less social/communicative dimensions such as eye color and hair color (Experiment 2). According to human genetic studies (e.g., Sulem et al., 2007; Sturm, 2009), high binding conditions were represented by combinations which have been reported to occur more frequently bound together, such as blue eyes-blonde hair, whereas low binding conditions were represented by combinations which have been reported to occur less frequently bound together, such as, e.g., blue eyes-brown hair.

## Materials and Methods

### Participants

Participants were undergraduates from the University of Pavia. Twenty took part in Experiment 1 (mean age = 23 years, SD = 2.54; 14 females) and 20 took part in Experiment 2 (mean age = 24.6 years, SD = 3.06; 16 females). None of them participated in both experiments. All volunteered in exchange for partial course credit, gave informed consent and were naïve as to the purpose of the experiment.

### Apparatus and materials

For both Experiment 1 and 2 a novel computerized updating task was devised starting from a previous one (see Artuso and Palladino, 2011). Stimuli were presented on a 17″ monitor driven by an Asus computer, located 60 cm from the observer. Stimulus presentation and response registration were controlled by the SuperLab software.

The stimuli were color photographs of faces selected from the Radboud Faces Database by Langner et al. (2010) and measured 5.6 × 6.8°. For Experiment 1 10 faces were selected (five female and five male) showing joyful, fearful, or neutral facial emotional expressions. A face with the neutral facial expression was always presented at the start of the trial to allow participants some time to adapt to the processing of a complex visual stimulus such as a human face (stimulus adaptation; see also below). Each facial expression was taken with gaze either direct or averted. Gaze direction and facial expression combinations were counterbalanced within faces. For Experiment 2 10 different faces were selected from the same database, displaying direct gaze, and neutral facial expression. Five were female and five male, and showed blue or dark eyes and blonde or brown hair. Each blonde/brown hair face was taken with eyes either blue or dark. As in Experiment 1, at the start of the trial there was an adaptation phase in which a face with the neutral facial expression displaying direct gaze and with a combination of eye-hair color in accordance with the binding trial (i.e., high or low, see below) was always presented. Eye and hair color combinations were counterbalanced within faces.

### Procedure and design for experiment 1

Participants were told that the experiment was composed of trials varying in length, randomized in the experiment. They were presented with a number of faces, displayed in the center of the screen one at a time. The number of the faces displayed varied from two to four faces. As suggested by updating literature which emphasizes the use of an unknown list length to have the participant process each incoming item (see Morris and Jones, 1990), we presented lists of different length, so as to increase task variability and to make the end of the list unpredictable to participants.

**Figure 1:**
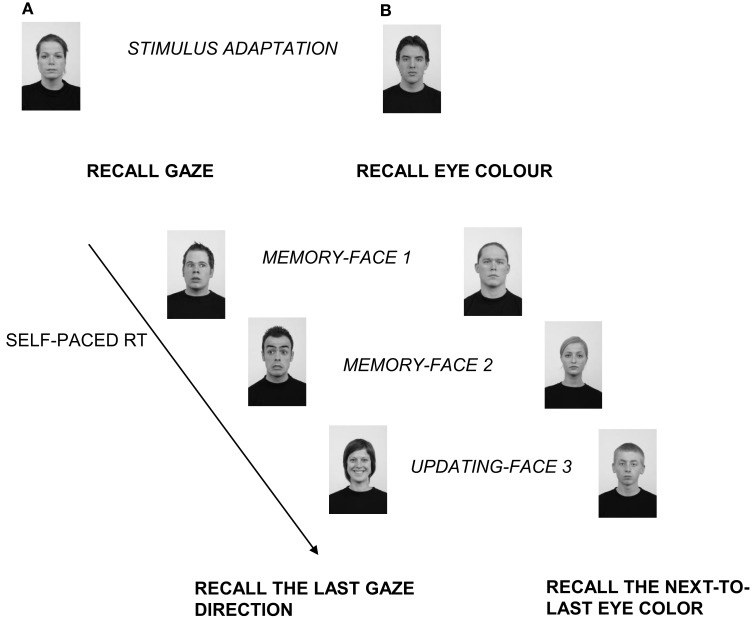
**An example of the sequence of events present in a trial requesting one update from Experiment 1 (A) and Experiment 2 (B)**. **(A)** After the initial stimulus adaptation phase, the participants were instructed to focus on and memorize the direction of the gaze of the face, disregarding the facial expression. After reading the instruction, they had to press the spacebar to see each face of the trial sequence. The self-paced experimental part of the trial started when the first face (Face 1, displaying here an averted gaze) appeared. Since the participants’ task was to recall at the end of the trial either the direction of the gaze or the facial expression of the last/next-to-last of the seen faces, they had to memorize the direction of the gaze of Face 1 and to keep it in mind. Then, when the participants pressed the spacebar, the second face (Face 2) was presented. Here again, the face showed an averted gaze. At that point, participants had in mind the gaze direction of the first two faces (averted–averted). Next, when the third face appeared displaying direct gaze, an updating phase took place. In it the participants had to substitute the direction of the gaze of the first memorized face with the most recent memorized one. Namely, they had to substitute the direction of gaze of Face 1 with the direction of gaze of Face 3, thus updating their representation. They now had in mind the new association of gaze direction: averted-direct. Finally, at the end of the trial, at the recall test phase, in this case they had to recall the most recent memorized gaze by pressing the key corresponding to direct gaze on the keyboard. **(B)** In Experiment 2 the sequence of events was the same as in Experiment 1 but the instruction request here was to focus on and memorize either eye color (shown here) or hair color. The stimuli here are reproduced in grayscale and are not drawn to scale.

Their task was to memorize the displayed faces and, in case, to compare and substitute the information of two consecutive faces, in terms of a specific facial dimension. At the end of the trial they were asked to recall the information of the last or next-to-last face. Therefore, in order to correctly perform the task they had to keep in mind two consecutive faces. Furthermore, during the task participants were instructed to respond as accurately and quick as possible, so that they were not encouraged to process the stimuli strategically. For half of the trials, participants received the specific instruction to focus on and memorize gaze direction disregarding facial expression; for the other half, to focus on and memorize the emotional expression, disregarding gaze direction. See Figure 1 for an example.

All the trials had the same procedure: an initial stimulus adaptation phase, two memory phases, one or two updating phases followed immediately by a recall test phase.

In the stimulus adaptation phase, a neutral face gazing straight ahead was presented. Then, the instruction on which facial dimensions the participant had to focus (gaze direction vs. facial expression) was presented. The participants had to press the spacebar to start the experimental part of the trial.

In the memory phases, participants had to study, memorize, and maintain the information about each face. Thus, after two memory phases, they had to maintain the information about two consecutive faces.

In the updating phase, after the first two studied faces, when a third face was displayed (and sometimes a fourth), the participant had to compare it (or them) with the previously studied faces and to substitute it when necessary, as exemplified by Figure 1A. This comparison/substitution process represented the updating function in terms of maintenance of information and inhibition of irrelevant information, through its replacement (see also Introduction).

At the end of each trial, according with the initial instruction, participants were asked to recall either gaze direction or facial expression of the last studied face or the next-to-last studied face. The recall request (which face had to be recalled) was displayed in the center of the screen. Participants responded by pressing on the computer keyboard one of two keys for gaze direction (“M” for direct gaze, “Z” for averted gaze), and another two for facial expression (“A” for a joyful face, “L” for a fearful face).

The task was self-paced, that is, participants pressed the spacebar to start each trial and after each phase of the trial (i.e., adaptation, instruction, memory, updating), in order to carry on with the task. This allowed the participants to study the stimuli at their own pace enabling a less biased performance. Classically, in updating literature the pace of the task is established by the experimenter. However, it has been observed that fixing stimulus presentation pace might create both recency and anxiety effects (e.g., Bunting et al., 2006; Palladino and Jarrold, 2008). To avoid this risk, we used a self-paced stimuli presentation. The RT for each of the memory and updating phases was collected as dependent variables. In addition, accuracy at the final recall test was measured.

After 16 practice trials, a total of 144 trials, divided in four experimental blocks, were presented individually to participants. The order of presentation of the blocks was counterbalanced across participants.

The variables of interest were Binding, Facial Dimension, and Phase. Binding was randomized between trials, whereas Facial Dimension was blocked, as participants received half of the blocks with the instruction to focus on gaze direction and the other half with the instruction to focus on emotional expression.

Binding represented the strength of the specific biological combinations of gaze direction and facial expression. High bindings were the combinations of gaze direction-facial expression (i.e., joy-direct gaze; fear-averted gaze) previously found to be perceived and processed strongly bound together. Low bindings were the combinations of gaze direction-facial expression (i.e., joy-averted gaze; fear-direct gaze) found to be perceived and processed weakly bound together. Facial Dimension represented the stimulus dimension (gaze direction or facial expression) which participants were instructed to memorize/update and to recall at the end of the sequence. The Phase variable corresponded to the different process involved in each specific phase of the task: memory or updating.

### Procedure and design for experiment 2

The procedure in Experiment 2 was exactly the same as in Experiment 1, except that the Facial Dimension of interest was represented by eye color or hair color, and thus the Bindings were different combinations of eye color-hair color. Specifically, high bindings were combinations which have been reported to occur strongly bound together (i.e., dark eyes-brown hair; blue eyes-blonde hair); whereas low bindings were combinations which have been reported to occur bound together more weakly (i.e., blue eyes-brown hair; dark eyes-blonde hair; see e.g., Sulem et al., 2007). At the recall test phase, participants had to press the same keys used in Experiment 1 except that now they indicated the two dimensions of eye color and hair color. Namely, participants responded by pressing keys on the computer keyboard (“M” for blonde hair, and “Z” for brown hair, and “A” for blue eyes, and “L” for dark eyes).

## Results

Only trials in which the recall was correct were analyzed in both Experiment 1 (97.78%) and Experiment 2 (93.36%). As we obtained high accuracy at recall, in line with previous findings (e.g., Artuso and Palladino, 2011), we focused our analysis only on task self-paced RTs, which have been shown to be much more sensitive to the updating process (see Kessler and Meiran, 2008; Artuso and Palladino, 2011). It is worth mentioning that the traditional updating measure is accuracy, which is used in the running memory span task (Morris and Jones, 1990) and similar tasks. However, it has been shown that accuracy (i.e., the number of correctly remembered items) tends to combine all the processes active during the task, collapsing them into a global index of memory efficiency, and to mask their separate contributions. Thus, in order to avoid this problem, and following some recent contributions to the update literature (e.g., Kessler and Meiran, 2008; Artuso and Palladino, 2011) we adopted the self-paced RT as a more direct measurement of the updating process.

### Data treatment

Response times exceeding individual participant means for each condition by more than three intra-individual standard deviations were considered outliers in both Experiment 1 (4.43%) and Experiment 2 (7.28%) and therefore excluded from the analyses. The RTs for each memory and updating phase were calculated as follows. At the memory phases, as well as the updating phase, the RT was computed starting from the onset of the first face until the participants (having memorized/updated the face) pressed the spacebar to continue the task and see the next face. Then, the mean RTs were computed for each specific phase. The mean RTs of the memory phases were computed averaging the RTs of all the memory phases of each trial. An analogous procedure was used to calculate the mean RTs of the updating phases.

### Experiment 1

Experiment 1 RTs were entered in an ANOVA with Binding (high, low) × Facial Dimension (gaze direction, facial expression) × Phase (memory, updating) as within-subjects factors. The inter-participant means of RTs are shown in Table 1.

**Table 1:** **Mean reaction times in ms and standard deviation (in brackets) from Experiment 1 subdivided for each condition**.

		High binding	Low binding
Memory	Gaze direction	576.58 (12.30)	669.52 (20.39)
	Facial expression	649.27 (3.71)	697.11 (11.89)
Updating	Gaze direction	778.88 (29.25)	837.38 (17.53)
	Facial expression	653.09 (22.18)	667.17 (9.40)

The main effects were all significant. The main effect of Binding, *F*(1, 19) = 206.01, partial **η^2^** = 0.98, *p* < 0.001, showed quicker RTs for high bindings (*M* = 664 ms; SD = 5.89 ms) than for low bindings (*M* = 717 ms; SD = 8.72 ms). The main effect of Facial Dimension, *F*(1, 19) = 176.21, partial **η^2^** = 0.90, *p* < 0.001, showed longer RTs for processing gaze direction (*M* = 715 ms, SD = 12.37 ms) than for facial expression (*M* = 666 ms, SD = 8.37 ms).

In addition, the main effect of Phase, *F*(1, 19) = 446.02, partial **η^2^** = 0.99, *p* < 0.001, showed that updating phases required longer RTs (*M* = 734 ms, SD = 8.60 ms) than memory phases (*M* = 648 ms, SD = 5.90 ms).

The two-way interaction between Binding and Facial Dimension was also significant, *F*(1, 19) = 80.97, partial **η^2^** = 0.81, *p* < 0.001. *Post hoc* planned comparisons, *t*(19) = 25.90, *p* < 0.001, showed a greater advantage for facial expression processing (vs. gaze direction) in low binding conditions, relative to high binding conditions, *t*(19) = 5.31, *p* < 0.001. A possible explanation for this result is that facial expressions may be treated as global configurations, whereas gaze direction is likely to be considered as a single component of a face, thus requiring a more analytical approach to be processed (see also Discussion).

**Figure 2:**
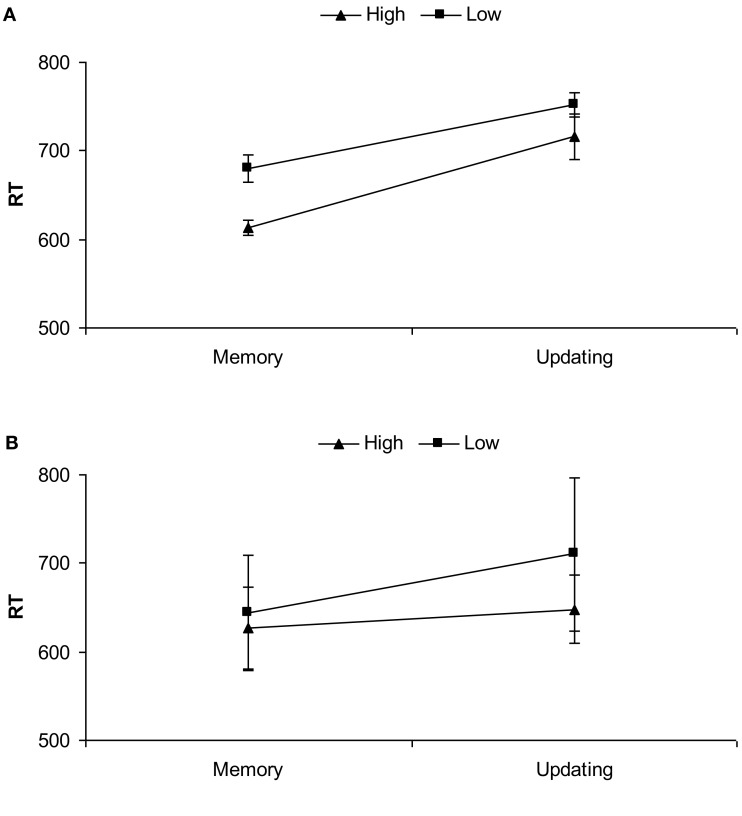
**Mean RTs (ms) and updating phases as a function of high and low biological bindings for Experiment 1 (A) and Experiment 2 (B)**. Bars show standard deviations.

The two-way interaction between Binding and Phase was significant as well, *F*(1, 19) = 36.28, partial **η^2^** = 0.66, *p* < 0.001. *Post hoc* planned comparisons showed that updating phases always required longer RTs than memory phases, both in high binding conditions, *t*(19) = 26.15, *p* < 0.001, and in low binding ones, *t*(19) = 24.16, *p* < 0.001. Moreover, although RTs in high binding conditions were faster compared to low binding conditions in the memory phase, *t*(19) = 19.12, *p* < 0.001, in the updating phase the advantage for high binding conditions was smaller, but still significant, *t*(19) = 13.52, *p* < 0.05. See Figure 2A.

The three-way interaction was not significant, *F* < 1.

### Experiment 2

Experiment 2 RTs were entered in an ANOVA with Binding (high, low) × Facial Dimension (eye color, hair color) × Phase (memory, updating) as within-subjects factors. The inter-participant means of RTs are shown in Table 2.

**Table 2:** **Mean reaction times in ms and standard deviation (in brackets) from Experiment 2 subdivided for each condition**.

		High binding	Low binding
Memory	Hair color	625.26 (49.24)	659.66 (66.28)
	Eye color	628.26 (42.84)	629.32 (64.40)
Updating	Hair color	654.24 (34.21)	745.26 (89.00)
	Eye color	642.45 (42.35)	674.68 (83.13)

The main effects were all significant. The main effect of Binding, *F*(1, 19) = 14.61, partial **η^2^** = 0.44, *p* < 0.001, showed quicker RTs for high bindings (*M* = 637 ms; SD = 28.70 ms) than for low bindings (*M* = 677 ms; SD = 67.08 ms). The main effect of Facial Dimension, *F*(1, 19) = 35.58, partial **η^2^** = 0.65, *p* < 0.001, showed longer RTs for processing hair color (*M* = 671 ms, SD = 44.72 ms) than for eye color (*M* = 643 ms, SD = 49.19 ms).

In addition, the main effect of Phase, *F*(1, 19) = 21.75, partial **η^2^** = 0.53, *p* < 0.001, showed that updating phases required longer RTs (*M* = 679 ms, SD = 53.67 ms) than memory phases (*M* = 635 ms, SD = 48.57 ms).

The two-way interaction between Binding and Facial Dimension was also significant, *F*(1, 19) = 101.11, partial **η^2^** = 0.84, *p* < 0.001. *Post hoc* planned comparisons, *t*(19) = 23.70, *p* < 0.001, showed a greater advantage for eye color processing (vs. hair color) in low binding conditions, relative to high binding conditions. The results can be accounted for by the fact that eyes are components of face which receive high priority in processing (see e.g., Henderson et al., 2005); consequently, they are processed faster and more efficiently than other face components.

The two-way interaction between Binding and Phase was significant as well, *F*(1, 19) = 21.68, partial **η^2^** = 0.53, *p* < 0.001. *Post hoc* planned comparisons showed that RTs in high binding conditions were faster than low binding conditions at the memory phase, *t*(19) = 18.22, *p* < 0.05, and this difference increased at the updating phase, *t*(19) = 21.43, *p* < 0.001. Moreover, updating phases always required longer RTs than memory phases, but less so in high binding conditions, *t*(19) = 11.23, *p* < 0.05 than in low binding conditions, *t*(19) = 22.35, *p* < 0.001. See Figure 2B.

Again, the three-way interaction did not reach significance, *F* < 1.

## General Discussion

The present study is original in terms of bringing together two distinct areas of research: WM updating and the perception of faces, in particular the processing of biological binding between facial dimensions. It provides an initial answer as to how the bindings between facial dimensions that have an important social and communicative value are updated; a function of WM that is important for guiding our social behavior.

The aim of the study was to investigate how the biological binding between different facial dimensions may impact updating processes in WM. Two new results emerged. First, in contrast to previous findings for non-biological bindings, faster processing times were found for the updating of combinations of facial dimensions highly bound together; second, our data showed that the ease of updating highly bound facial dimensions varied depending on the communicative and social meaning of the binding to be updated.

In terms of the first result, our data indicate an advantage in the updating of high biological binding. Thus, WM updating seemed to benefit when different facial dimensions were highly bound together. This is a new and original result, not evident in the existing updating literature, which has focused on other kinds of bindings, i.e., non-biological bindings, arbitrarily created, such as between letters or digits. In fact, previous findings showed that when stimuli with no social/biological value were highly bound, their binding had a negative impact on WM performance (e.g., Guérard et al., 2009; Artuso and Palladino, 2011). Indeed, when the binding was based on phonological or perceptual similarity, high bindings required longer RTs to be dismantled, updated and re-created. Conversely, in the present study, where biological bindings were considered, the stronger they were combined the faster they were updated. This advantage is likely to be due to the fact that faces are important stimuli for social and interpersonal behavior. Therefore, when living in a social world these stimuli need to be updated quickly in order to be beneficial for our social behavior. These results are important because they suggest that updating processes support face processing in a way that is consistent with keeping track of the communicative value of facial dimension bindings. In this respect, the inclusion in our study of non-biological bindings would not have been informative of the effect of social and communicative salience of the binding on WM updating.

An alternative explanation is also possible. High biological bindings are not updated faster to allow the tuning of WM to an ongoing social environment, rather, their faster updating is a consequence of being well-learned, due to their enhanced perceptual processing (e.g., Adams and Kleck, 2003). In other words, it is thanks to a learning process that they have a more rapid access to WM. Therefore, these highly bound combinations could just work more effortlessly, more fluently, than the processing of weakly bound combinations. If this is the case, then WM updating would benefit indirectly by a faster analysis of the relevant stimulus dimension. However, although plausible and worthy of further research, this explanation is unlikely and should be regarded with some caution. In fact, the results on the updating of well-learned bindings with stimuli having no biological value (e.g., 12 taken as a well-learned memory binding) indicate that the strength of this binding was not enough to produce an advantage in updating (Artuso and Palladino, 2012).

A related issue is also worth mentioning. Different kinds of bindings do exist (e.g., Piekema et al., 2010; see also Ecker et al., 2012). In the present study, we focused our investigation on the updating of the binding between two dimensions of the same kind of stimulus, that is the face. However, in the traditional updating literature, usually the bindings between different stimuli (e.g., letter–letter) are investigated. Thus, the advantage we found in the updating of high binding conditions might also be ascribed to the different type of binding, i.e., of different dimensions of the same stimulus. To clarify this point, further studies will be needed to separate these different bindings and their biological value.

The present results show that the processing of facial expression was faster than that of gaze direction, particularly in low binding conditions (see Experiment 1, Results). A possible explanation, as mentioned earlier, is that facial expressions may be treated as global configurations, whereas gaze direction is likely to be treated as a single component of a face. This is in accordance with neurophysiological results which show that the processing of the internal parts of a face, such as gaze direction, is slower than the processing of the same overall face (see e.g., De Souza et al., 2005). Conversely, in high binding conditions gaze direction and facial expression seem to have been processed in a more global or holistic way given that the difference between gaze direction and facial expression was significantly reduced.

Closely related to the updating processing, we found that the updating phases showed longer latencies than the memory phases. Thus, the updating phase was clearly different from the memory phase (i.e., longer latencies; see both Experiments but especially Experiment 1), as it required more effort to complete. This is in accordance with the traditional conceptualization of updating and is also consistent with previous findings which characterize memory and updating as distinct cognitive processes (e.g., Morris and Jones, 1990; Palladino and Jarrold, 2008; Artuso and Palladino, 2011). Indeed, when the memorized information became no-longer relevant and had to be substituted with new relevant information, this was more difficult than just the memorization of information. Thus, the results suggest that the task we devised was suitable and effective in distinguishing the memorization process from the updating process. In turn, it contributes to memory updating literature, by showing that similar processing effects can be found across different stimuli manipulations. Moreover, consistent with previous findings (e.g., Morris and Jones, 1990; Palladino et al., 2001), we found an effect of the number of updates, consisting of an increase in response latencies with the number of requested updates^1^

In addition, the fact that highly bound combinations had shorter latencies in memory phases, as well as in updating phases confirms that participants based their response on perceptual processing, combining the facial dimensions of the faces (i.e., gaze direction-facial expression and hair color-eye color), rather than verbalizing it. In other words, the fact that we found an advantage for high binding conditions at the memory phase as well as at the updating phase indicates that these conditions required less effort to encode and memorize. Moreover, the advantage found at the updating phase could not be based on a verbalization of facial dimensions, which would otherwise have produced the same effect on both low and high binding conditions.

In terms of our second result, although we found an overall advantage for the updating of high binding conditions, compared to memory, our data also suggests that the two bindings affected updating differently. Interestingly, for the highly bound combinations of gaze direction and facial expression, we found a large difference in response latencies between the memory and updating phases. On the contrary, for the binding between eye color and hair color, memory and updating phases were still different across high binding conditions, but their difference was reduced. In other words, our data shows that the binding between the dimensions which are genetically determined and having less communicative value (i.e., eye color-hair color) required less effort to update, since the difference between the study and updating phases was very small. In contrast, the updating of the binding between the two changeable and more communicative dimensions of gaze direction and facial expression was clearly more difficult, relative to the memory phase. This was a new and unexpected result, as one might have expected gaze direction and facial expression to be rapidly and efficiently updated given that they are facial aspects which can change quickly, and that their different combination have different communicative meanings (see Adams and Kleck, 2003; N’Diyae et al., 2009). Therefore, to respond promptly and adapt our behavior to their change, one would expect our cognitive system to be able to update them very quickly.

We believe this result is interesting and we think this difference can be a consequence of the fact that the two high bindings require different updating processes. In particular, we might hypothesize that different cognitive processes come into play. For the binding between gaze direction and facial expression it is plausible that beyond studying the faces, memorizing and maintaining the relevant information, and updating their binding, participants also have to update the meaning of that binding. The meaning of the binding being its appraisal, i.e., the relevance of the stimulus to the observer (e.g., Mumenthaler and Sander, 2012). For example, if while the observer is looking at a happy face gazing straight ahead, the face suddenly becomes scared and moves the eyes toward a specific portion of space, the observer has to detect this change in gaze direction together with the emotion expressed, thus creating a new representation of the seen face, that is to update it. However, a second operation is also necessary: an updating of the appraisal of the seen face. In fact, the observer has to modify not only the combination between the two facial dimensions, but also the value and the meaning of that combination. This is particularly so, if the face which changes gaze direction and facial expression is also a new face as in the present study. So, if for instance the observer moves from a potentially pleasant stimulus to one which indicates a potential danger, she/he has to change the meaning of that face as well, in other words to update its ongoing cognitive appraisal.

In contrast, updating the color of the eye and the hair when highly bound together would require less or different cognitive processes. In fact, they appear easier and require less effort to update, as the updating phase latencies were not much longer than those of the memory phase. This might be because participants just have to study the faces, memorize, and update the relevant information, and update their binding, but no update of their meaning is probably necessary. In fact, these facial dimensions are less communicative and consequently they are not very relevant for the ongoing social and appraisal processes. Therefore, the observer is probably not continuously engaged in updating their meaning and their value for him/herself. This would explain why these combinations can be updated quickly.

Thus, the greater cost observed in the updating of highly bound combinations of gaze direction and facial expression, relative to their memorization (see Experiment 1), might reasonably be interpreted in terms of the amount and type of processes requested: the updating of the binding as well as the updating of the appraisal of that binding. Whereas, for the updating of highly bound combinations of eye and hair color, the updating of their meaning would not be needed, thus explaining the smaller cost of their updating compared to their memorization (see Experiment 2).

In low bound combinations, the differences were the same across the two kind of bindings (i.e., gaze direction-facial emotion and eye color-hair color) indicating that similar cognitive processes were taking place.

Therefore, it seems plausible that a cost in terms of cognitive processing which would be due to processing more social information, such as that found for the updating of highly bound gaze direction and facial expression, may translate eventually into an advantage for social cognition, because through the updating of the appraisal of a biological and highly communicative binding a more adaptive response to the environment can be given. Further investigations will be needed to corroborate our new results and to test this explanation.

Our results refer not only to the role of social stimuli in WM processes but are also informative about social cognition processes. Social cognition “refers to the processes that subserve behavior in response to conspecifics and in particular to those higher cognitive processes subserving the extremely diverse and flexible social behaviors seen in primates” (Adolphs, 1999, p. 469). Thus, to successfully live in a social environment, people must possess invariant representations of the immediate world but also be responsive to the unexpected changes, in particular changes in the behavior of others and in person perception: all aspects relevant for social cognition (see Macrae and Bodenhausen, 2000). This simultaneous stability and flexibility are likely to characterize social functioning as well as WM functioning. However to date, and to the best of our knowledge, only the study by Meyer et al. (2012) has specifically investigated the relationship between social cognition and WM. In a fMRI study, the authors, investigated which areas are recruited in a social WM task, i.e., mentalizing. Their results brought evidence for a specific social WM system which is recruited when we deal with social information. Therefore, they conclude by claiming that the purpose of social WM is to build-up and maintain an internal model of the immediate social environment and social world.

Our study resembles that of Meyer and co-workers in the sense that we also investigate how WM deals with pieces of social information, and in particular how WM is engaged in updating the faces of other people. As with mentalizing (see Meyer et al., 2012), face perception is thought to represent a building block of human social cognition.

Moreover, in line with the idea that the purpose of social WM is to keep track of the various social information which are crucial to navigating our social world and understand social interaction, our study is important because offers some first evidence of how the bindings between facial dimensions are updated. In particular, it suggests that the increase in social information conveyed by the binding with higher social and communicative value demands more WM resources to be updated. A possible reason for this is the necessity to also include in our internal model of the changeable social environment the immediate social or survival relevance of the stimulus to the observer. When perceiving faces this also results in being able to understand other people’s intentions and mental states. Moreover, this is in line with the proposal that memory for another individual’s face partly depends on an evaluation of the behavioral intention of that individual (Nakashima et al., 2012).

In conclusion, the present study indicates for the first time that updating of biological bindings benefits from the strength of their bindings. In addition, our study suggests that the ease of the ongoing updating processing in WM varies depending on the meaning of the facial dimension bindings that have to be updated, thus revealing a crucial role of WM updating in social cognition and appraisal processes.

## Conflict of Interest Statement

The authors declare that the research was conducted in the absence of any commercial or financial relationships that could be construed as a potential conflict of interest.

## Footnotes

^1^Because it was beyond the scope of the present study, analyses involving the Number of Updates (or Load) as a factor were not reported in the main text. However, to relate our results to the existing literature we ran two further analyses including Number of Updates as a factor. For both Experiment 1 and Experiment 2 we entered RTs in an ANOVA with Binding (high, low) × Facial Dimension × (gaze direction, facial expression) × Phase (memory, updating) × Number of Updates (0, 1, 2) as within-subjects factors. For Experiment 1, as well as finding the same results as those reported in the main text (in which the Number of Updates factor was collapsed), we also found a significant main effect of Number of Updates, *F*(2, 38) = 15.03, partial η^2^ = 0.44, *p* < 0.001. Means comparison, *t*(19) = 22.31, *p* < 0.001, showed longer RTs for processing trials requesting two updates, compared to trials with one update, and trials with no update (respectively, 664 vs. 643 vs. 636 ms). Similar results were found for Experiment 2. In particular, the main effect of Number of Updates was also significant, *F*(2, 38) = 4.41, partial η^2^ = 0.18, *p* < 0.01. Means comparison, *t*(19) = 15.22, *p* < 0.01, showed longer RTs for processing trials requesting two updates, compared to trials with one update, and trials with no update (respectively, 632 vs. 618 vs. 604 ms). Overall, the results show that RTs increased with the number of requested updates. This effect is consistent with the updating literature (see e.g., Morris and Jones, 1990; Palladino et al., 2001) and offers further support to the reliability of our findings and the validity of our task.
